# Supplementary material for: Evidence for Auditory Stimulus‐Specific Adaptation But Not Deviance Detection in Larval Zebrafish Brains
Source: J Comp Neurol. 2025 Mar 26;533(4):e70046. doi: 10.1002/cne.70046 (PMC11946781; doi:10.1002/cne.70046)
Supplement: Supplementary file 1 — Figure S1 Auditory supplemental information. Figure S2 Stimulus‐specific adaptation and oddball supplemental information. [file CNE-533-e70046-s001.pdf]

SUPPLEMENTAL INFORMATION FOR:

EVIDENCE FOR AUDITORY STIMULUS-SPECIFIC ADAPTATION BUT NOT DEVIANCE DETECTION IN LARVAL ZEBRAFISH BRAINS.

Maya Wilde[1,2], Rebecca E. Poulsen[3], Wei Qin[2], Joshua Arnold[1], Itia A. Favre-Bulle[1,4], Jason B. Mattingley[1,5], Ethan K. Scott[1,2, ‡], Sarah J. Stednitz[2, ‡].

1. Queensland Brain Institute, University of Queensland

2. Department of Anatomy and Physiology, University of Melbourne

3. Department of Linguistics, Faculty of Medicine, Health and Human Sciences, Macquarie University

4. School of Mathematics and Physics, University of Queensland

5. School of Psychology, University of Queensland

‡ Corresponding authors: sarah.stednitz@unimelb.edu.au, ethan.scott@unimelb.edu.au

SUPPLEMENTAL FIGURES & LEGENDS:

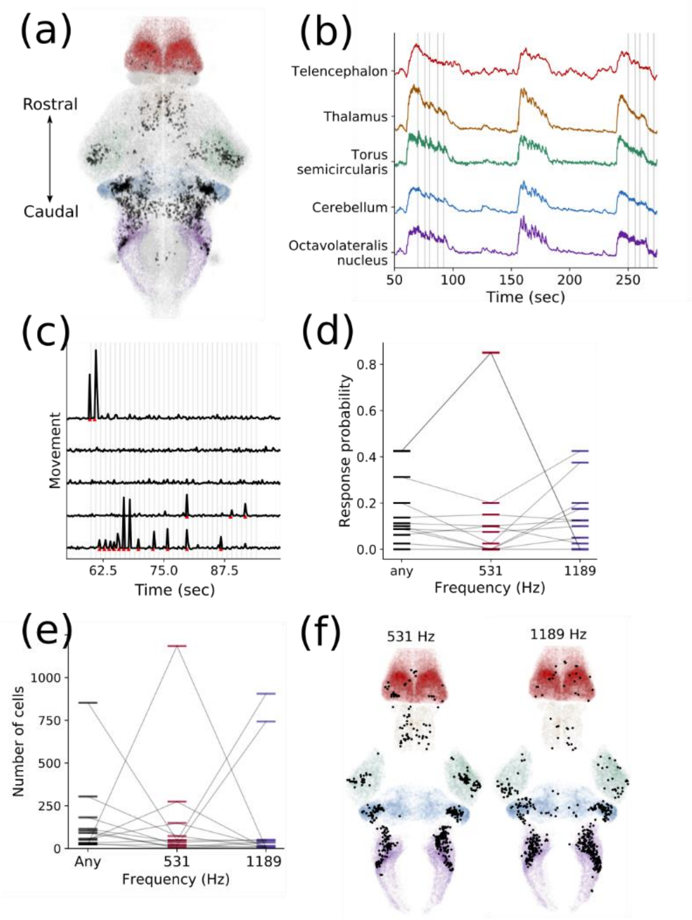

Figure S1: Auditory supplemental information. A) Distribution of auditory neurons, with auditory regions highlighted. The auditory neurons (black) almost entirely fall within the octavolateralis nucleus (purple), cerebellum (blue), torus semicircularis (green), thalamus (orange), and telencephalon (red). **B)** Average ΔF/F across brain regions implicated in the auditory processing pathway. Vertical lines indicate oddball tone presentations. **C)** Inferred fish movement for 5 example animals. Vertical lines represent stimulus presentations, red arrows indicate detected movement. **D)** Behavioural response probability (as inferred from motion correction) to any tone, 531 Hz, or 1189 Hz across all animals. Each line represents one fish. **E)** Contributions of each fish to generally auditory responsive cell populations (black), 531 Hz - preferring neurons (pink), and 1189 Hz – preferring neurons (purple). Each line represents a fish. All fish contribute to each group, but to different extents. **F)** Distributions of frequency-specific auditory neuron populations overlaid with outlines of auditory brain regions: octavolateralis nucleus (purple), cerebellum (blue), torus semicircularis (green), thalamus (orange), and telencephalon (red).

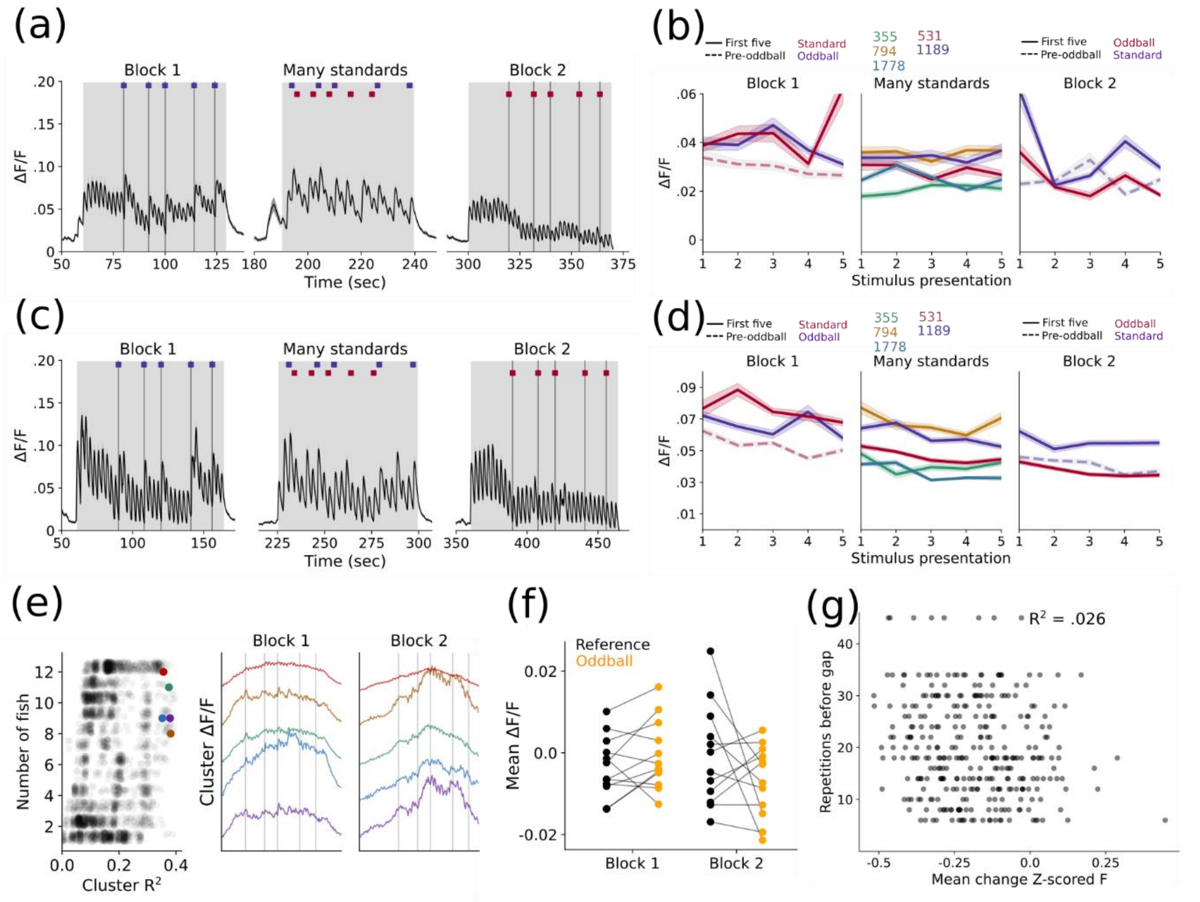

Figure S2: Stimulus specific adaptation and oddball supplemental information. A) Mean activity trace of all auditory responsive neurons in a dataset with a 2 second interstimulus interval. **B)** Mean response amplitudes of all auditory-classified neurons to specific stimulus presentations in a 2 second interstimulus interval dataset. In block 1 and 2, solid lines represent the first 5 presentations of each frequency (as either standard or oddball), and the dotted line represents the 5 presentations of the standard stimulus directly preceding an oddball. In the many standards block, the mean responses to 5 presentations of each frequency are shown. Shading represents standard error of mean. **C-D)** Same as A and B but for a dataset with a 3 second interstimulus interval. **E)** Left: clusters identified by 100 permutations of k-means clustering with 30 clusters, according to their representation across fish and correlation to the oddball stimulus. Right: Mean traces of example clusters highlighted on the right with high correlation to oddball stimuli. These activity traces do not resemble oddball-specific responses. **F)** Comparison of amplitude of responses from neurons in pooled clusters with $R^2 > 99^{th}$ percentile to oddball stimuli (yellow) and preceding standard stimuli (black) in each block. **G)** Mean ΔF/F by number of preceding sounds in the silent gap dataset. The magnitude of the dip at the time of the gap is not affected by the number of preceding sounds (R^2 = 0.026).
